# Short-Time Transport Properties of Bidisperse Suspensions of Immunoglobulins and Serum Albumins Consistent with a Colloid Physics Picture

**DOI:** 10.1021/acs.jpcb.2c02380

**Published:** 2022-09-16

**Authors:** Christian Beck, Marco Grimaldo, Hender Lopez, Stefano Da Vela, Benedikt Sohmen, Fajun Zhang, Martin Oettel, Jean-Louis Barrat, Felix Roosen-Runge, Frank Schreiber, Tilo Seydel

**Affiliations:** † Institut für Angewandte Physik, Universität Tübingen, Auf der Morgenstelle 10, 72076 Tübingen, Germany; ‡ Institut Max von Laue—Paul Langevin (ILL), CS 20156, F-38042 Grenoble Cedex 9, France; § School of Physics and Optometric & Clinical Sciences, Technological University Dublin, D07 XT95 Grangegorman, Ireland; ∥ Institut für Angewandte Physik, Universität Tübingen, 72076 Tübingen, Germany; ⊥ Univ. Grenoble Alpes, CNRS, LiPhy, 38000 Grenoble, France; # Department of Biomedical Science and Biofilms-Research Center for Biointerfaces (BRCB), Malmö University, 20506 Malmö, Sweden

## Abstract

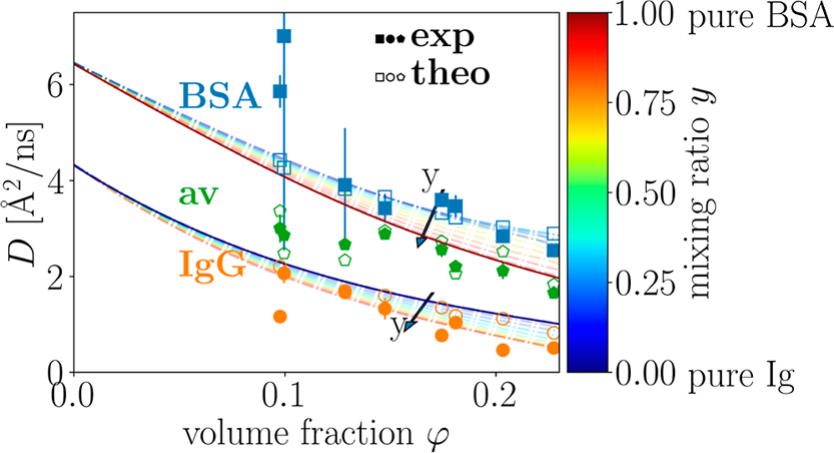

The crowded environment of biological systems such as the interior of living cells is occupied by macromolecules with a broad size distribution. This situation of polydispersity might influence the dependence of the diffusive dynamics of a given tracer macromolecule in a monodisperse solution on its hydrodynamic size and on the volume fraction. The resulting size dependence of diffusive transport crucially influences the function of a living cell. Here, we investigate a simplified model system consisting of two constituents in aqueous solution, namely, of the proteins bovine serum albumin (BSA) and bovine polyclonal gamma-globulin (Ig), systematically depending on the total volume fraction and ratio of these constituents. From high-resolution quasi-elastic neutron spectroscopy, the separate apparent short-time diffusion coefficients for BSA and Ig in the mixture are extracted, which show substantial deviations from the diffusion coefficients measured in monodisperse solutions at the same total volume fraction. These deviations can be modeled quantitatively using results from the short-time rotational and translational diffusion in a two-component hard sphere system with two distinct, effective hydrodynamic radii. Thus, we find that a simple colloid picture well describes short-time diffusion in binary mixtures as a function of the mixing ratio and the total volume fraction. Notably, the self-diffusion of the smaller protein BSA in the mixture is faster than the diffusion in a pure BSA solution, whereas the self-diffusion of Ig in the mixture is slower than in the pure Ig solution.

## 1 Introduction

Understanding the diffusive transport of macromolecules in the polydisperse and crowded ensemble within the aqueous intracellular fluid of living cells is crucial to understand their function.^1,2^ Polydispersity^3−5^ and crowding^6−12^ are, thus, subject to numerous theoretical,^13^ simulation,^11,14,15^ and experimental studies. Both living cells or even small organisms in their entire complexity^16−20^ and settings with different degrees of simplification have been explored using spectroscopic methods, such as nuclear magnetic resonance (NMR),^21,22^ Mössbauer spectroscopy,^23^ and fluorescence correlation spectroscopy.^24−26^ Drastically simplified model systems with well-defined and adjustable configuration parameters can help to test models, many of which are derived from colloid physics.^27−29^

To this end, aqueous solutions consisting of a single monodisperse protein species have previously been probed, inter alia, using neutron spectroscopy and NMR.^21,22,30−32^ On the diffusive short-time scale, direct interactions such as protein–protein collisions are negligible, and hydrodynamic interactions dominate which are considered crucial for biological function^33^ and for the quantitative understanding of long-time diffusion.^34−37^ On this short-time scale, it has been found that the self-diffusion (synonymously: tracer diffusion) of well-folded proteins with a compact shape can be quantitatively understood in terms of predictions for monodisperse colloidal hard spheres.^31^ Notably, the slowing down of the protein self-diffusion with increasing protein volume fraction in the suspension, that is, with increasing crowding, has been quantitatively understood for this situation. However, in actual biological systems, polydispersity is prevalent, so it is imperative to check the validity of the colloid picture in a genuinely polydisperse situation.

In this colloid picture, Stokesian dynamics simulations of binary hard spheres show that the larger component diffuses slower and the smaller component diffuses faster in a mixture than in their respective pure systems at the same total volume fraction.^38^ Realistic polydisperse simulations suggest that these rather complex systems can be mapped and understood by studying equivalent polydisperse hard-sphere models.^33^ Experimentally, the diffusion of immunoglobulin tracer proteins has recently been studied in such a naturally polydisperse and crowded setting of macromolecules^39^ and in dense phases after macroscopic phase separations.^40^ Simulations adapted to the particular polydisperse system^39^ generalize the trend seen in binary systems.^38,39^ Notably, particles smaller than the average effective radius of the macromolecular ensemble diffuse faster, and particles with a larger than average radius diffuse slower than in the monodisperse case at the same volume fraction. Computational studies showed similar effects influencing the self-diffusion close to interfaces.^4^ However, the existing explicit comparisons between experiment and colloid theory do not sufficiently address the genuine effects of polydispersity, for example, the experimental results in ref (39) can still be approximately described by an effective monodisperse system due to the experimental restriction to only one type of tracer protein.

Hence, a dedicated study of a model polydisperse system with tunable tracer composition is necessary, which in its simplest case would be a bidisperse system with a tunable total volume fraction and relative composition. Up to now, the separation of different diffusive contributions was possible either due to partial deuteration of the samples investigated^39^ or to the use of advanced modeling in hydrated powders.^41^ Here, we test the predicted bulk behavior of an aqueous solution of two distinct tracer proteins, namely, bovine serum albumin (BSA) and bovine polyclonal gamma-globulin (Ig). We separate the apparent center-of-mass diffusion coefficients measured simultaneously for both proteins from the internal diffusive processes and the solvent contributions. We use both proteins in their native, protonated form and vary the protein volume fraction and ratio. This simplified setting, compared to the highly polydisperse mixtures previously studied, allows for a more quantitative comparison with results from simulations. It also circumvents the need for biological deuteration. Thus, by analyzing the data employing different frameworks, we show the feasibility to investigate the short-time diffusive processes of two distinct species of label-free tracers in a solution and to separate and analyze the corresponding contributions to the scattering signal of the two proteins.

## 2 Materials and Methods

### 2.1 Sample Preparation

Polyclonal Ig (G5009) (*M*_Ig_ = 150 kDa^42^), BSA (A3059) (*M*_BSA_ = 66.4 kDa^43^), and D_2_O were purchased from Sigma-Aldrich (now Merck KGaA) and used with no further purification. The samples were prepared by dissolving given masses of the proteins in D_2_O as described in earlier studies.^44,45^ The sample details can be found in Table S1.

### 2.2 Quasielastic Neutron Spectroscopy Measurements

For the quasielastic neutron spectroscopy (QENS) measurements, the samples were filled in double-walled cylindrical aluminum sample containers with a 23 mm outer diameter and a gap between the walls containing the sample fluid with a width of 0.15 mm. The containers were sealed with indium wire.

The QENS spectra were measured during the experiments 9-13-526^46^ and 8-04-759^47^ on the neutron backscattering spectrometer IN16B^48^ at the Institut Max von Laue—Paul Langevin (ILL) in Grenoble, France. For calibration, an empty cylinder, pure D_2_O, and vanadium were additionally measured. IN16B was used with Si(111) monochromator and analyzer crystals, corresponding to the elastic wavelength λ = 6.27 Å. The investigated *q*-range (0.2 Å^–1^ ≤ *q* ≤ 1.9 Å^–1^) corresponds to nanometer length scales. A phase space transformation chopper enhanced the neutron flux at the sample position at the expense of an acceptable beam divergence.^49^ A standard Orange cryofurnace was employed to set the sample temperature.

### 2.3 Data Reduction

The IN16B data were reduced and analyzed using MATLAB. The empty can contribution was subtracted from all samples measured. Vanadium spectra were fitted for each momentum transfer *q* value with a sum of two Gaussian functions to analytically account for the instrument resolution in the subsequent fits of the sample spectra. The solvent contribution was fixed based on the pure D_2_O measurements following the approach explained in ref (44) using the total protein volume fraction φ. Further analysis of the diffusion coefficients was performed with python3^50^ using Jupyter Notebooks.

### 2.4 Analysis of the Scattering Signal with a Simplified Approach

For the investigated range in the energy transfer ℏω and in the momentum transfer *q*, the measured scattering function is dominated by the incoherent scattering signal *S*(*q*,ω) of the protein solution and can be written as the convolution of the resolution function  and the weighted sum of the contributions from the solvent (44) and from the protein *S*_Protein_(*q*,ω)^51^
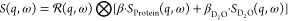
1where β(*q*) and  are scalar parameters. For globular proteins, the signal arising from the diffusive protein motions can be separated into an apparent global center-of-mass *S*_glob_(*q*,*ω*) and internal *S*_int_(*q*,*ω*) contribution
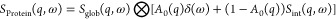
2with *A*_0_(*q*) being the elastic incoherent structure factor (EISF).^52^

Previous studies investigated the dependence of the apparent short-time center-of-mass diffusion coefficients on the protein volume fraction for several proteins in single-component solutions.^31,44,45^ For different globular proteins, an apparent diffusion coefficient *D* = *D*(*D*_r_,*D*_t_), consisting of the translational *D*_t_ and rotational *D*_r_ diffusion, has been observed for significantly changing environments. For the time and length-scale investigated, it can be described by a Fickian diffusion process, which translates into a Lorentzian function  with the width γ(*q*) = *Dq*^2^.^40,45,51,53,54^ In the energy transfer range investigated, the internal diffusive contribution of the proteins can also be described by a Lorentzian function.^45,51^ The incoherent scattering signal can thus be approximated by
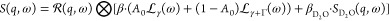
3

with  and  describing the apparent global diffusion and the internal diffusive processes, respectively. Although this approach is developed for the monodisperse system, several studies have shown that it can be applied to cluster forming systems.^45,53^ In this case, eq 3 is averaging, on the one hand, over the different global diffusive dynamics of the two proteins and, on the other hand, over their internal dynamics.

### 2.5 Separation of the BSA and Ig Contributions in the Scattering Signal

For *n* different proteins in the solution, the total incoherent scattering signal from the proteins *S*_∑_(*q*,*ω*) can be written as a weighted sum of the different protein contributions.
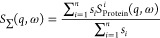
4with *S*_Protein_^*i*^(*q*,ω) being the scattering signal of the protein *i*. The incoherent scattering cross-section *s*_*i*_ of this protein is calculated as the sum *s*_*i*_ = *n*_p_ ∑_*j*_σ_*j*_ of the incoherent scattering cross-sections σ_*j*_ of the atoms present in this protein multiplied by the number of this type of protein *n*_p_ in solution.

To extract the individual diffusion coefficients of BSA and Ig separately from the experimental data, an advanced algorithm for fitting both the *q* and ℏ*ω* dependence simultaneously has to be applied to avoid overfitting due to the spectrometer resolution, limited energy transfer, and statistical errors of the measured spectra. The fit according to eq 3 has to be changed to
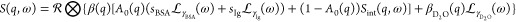
5with γ_BSA_ = *D*_exp_^(BSA)^*q*^2^ and γ_Ig_ = *D*_exp_^(Ig)^*q*^2^ and *D*_exp_^(Ig)^ < *D*_exp_^(BSA)^. The scaling parameters *s*_BSA_ and *s*_Ig_ are calculated using 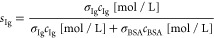
6and
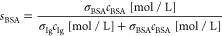
7where σ_Ig_ = 1011495.41 barn and σ_BSA_ = 464377.95 barn are the incoherent scattering cross sections of Ig and BSA, calculated based on the pdb files 1IGT^55^ and 4F5S (biological assembly 1),^56^ respectively.^57,58^*c*_Ig_ [mol/L] and *c*_BSA_ [mol/L] are the molar concentrations of Ig and BSA, respectively. The internal contribution  described by a Lorentzian function and the EISF *A*_0_, fitted as the *q*-dependent parameter with the limitation to be monotonically decreasing, were averaged over both proteins. The very high energy resolution of IN16B is optimal to accurately determine the protein center-of-mass diffusion, which comes at the cost of a limited energy range. It has to be emphasized that this limited energy transfer available and the apparent background given by the solvent do not allow us to separate the internal contributions of both proteins. Therefore, a simplified model is used for separating the apparent diffusion coefficients *D*_exp_^(BSA)^ and *D*_exp_^(Ig)^ of BSA and Ig, respectively, but keeping one single Lorentzian function averaging over the internal dynamics of BSA and Ig. The width of this Lorentzian function is chosen to be larger than the width of the Lorentzian function describing the apparent diffusion of BSA. Since the fit averages over the different contributions of the internal diffusion of BSA and Ig and over the corresponding EISFs, a detailed analysis of the internal dynamics based on this data set is not possible. The goodness of fit is slightly better for the model accounting for the two distinct protein species, compared to the average population model (cf. Figure S3). This improved goodness of fit and the prior knowledge, due to the sample preparation, of the existence of the two protein species therefore justify the application of the latter model. It should be emphasized that the number of fit parameters for the bidisperse model is increased by just one relative to the model describing only the average center-of-mass diffusion. This number of fit parameters in the fit applied to all *q* simultaneously is still lower than the number of fit parameters applied in the model-free approach using two free Lorentzian functions for each *q*. By investigating the dependence of χ_ν_^2^ as a function of the optimization parameters, the robustness of the fit can be evaluated. For the two different global fit approaches, these dependencies are shown in Figure S4.

### 2.6 Calculation of the Theoretical Diffusion Coefficients

Computational studies on multicomponent solutions indicate that the diffusion coefficient of a tracer changes due to the presence of a second type of tracer particle.^38,39^ Therefore, to compare the experimental and simulation results for our two-component system, we use the model reported by Wang and Brady^38^ for the short-time diffusion of bidisperse suspensions of hard spheres. Specifically, we employed the reported polynomial expressions for the diffusion coefficients (for both translational and rotational). These expressions depend on the volume fraction and the composition of the mixture and are based on pairwise additive approximation (two body interactions) and semi-empirical formulas (more details can be found in the Supporting Information). For each sample condition (i.e., for a given composition and volume fraction), the translational diffusion coefficient *D*_t_ and rotational diffusion coefficients *D*_r_ were calculated using the volume fraction  obtained employing the number density *n*_*i*_ = *c*_p_ [mg/mL]/*M*_W_^*i*^·*N*_A_, the molecular weight *M*_w_^*i*^, the hydrodynamic radius *R*_H_^*i*^ for protein *i*, and the Avogadro constant *N*_A_. The apparent center-of-mass diffusion coefficient *D* is calculated subsequently using the implicit relation for *D* = *D*(*D*_r_,*D*_t_)^31^ (see the Supporting Information for details).

We use the relative deviation of the apparent center-of-mass diffusion coefficients *D*_theo_(ϕ_theo_) from the monodisperse case (*y* = 0 and *y* = 1 for pure Ig and BSA, respectively) and multiplied by the corresponding experimental volume fraction dependence determined previously^31,44^ to obtain the theoretical apparent diffusion coefficients *D*_theo_(*φ*)
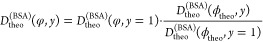
8
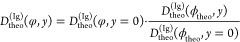
9

This calculation is necessary, since the direct conversion between the experimentally given volume fraction φ of the mixture and the effective hydrodynamic volume fraction ϕ_theo_ determining the theoretical diffusion is only possible for the pure solutions^31,44^ but not for mixtures.

By following this pathway, it is possible to express the simulation results in terms of the experimental conditions. In addition, by performing this renormalization, a possible presence of dimers of BSA^59−61^ and Ig^62^ and slow domain motions of Ig, which are captured by the thinner Lorentzian function,^40^ are taken into account. We note that a fraction of BSA or Ig dimers might be present in the samples, but the picture of monomers is sufficient to model the results. The effect of possible oligomers might cancel out in the comparison due to the scaling of the theory to the effective hydrodynamic size of the proteins.

## 3 Results and Discussion

### 3.1 Experimental Average Center-of-Mass Diffusion

To investigate the short-time self-diffusion of Ig and BSA in the same solution, we employ QENS using IN16B,^48^ as this technique proved to be well-suited for the study of the nanosecond protein dynamics at high protein volume fractions^31,63^ (cf. Section 2.4).

In a first, simpler approach, the QENS signal is analyzed for each recorded value of the momentum transfer ℏ*q* individually. Based on eq 3, the scattering signal of the two proteins was described by one Lorentzian function averaging over the center-of-mass diffusion and a second Lorentzian function averaging over the internal diffusive processes in the two proteins.

**Figure 1:**
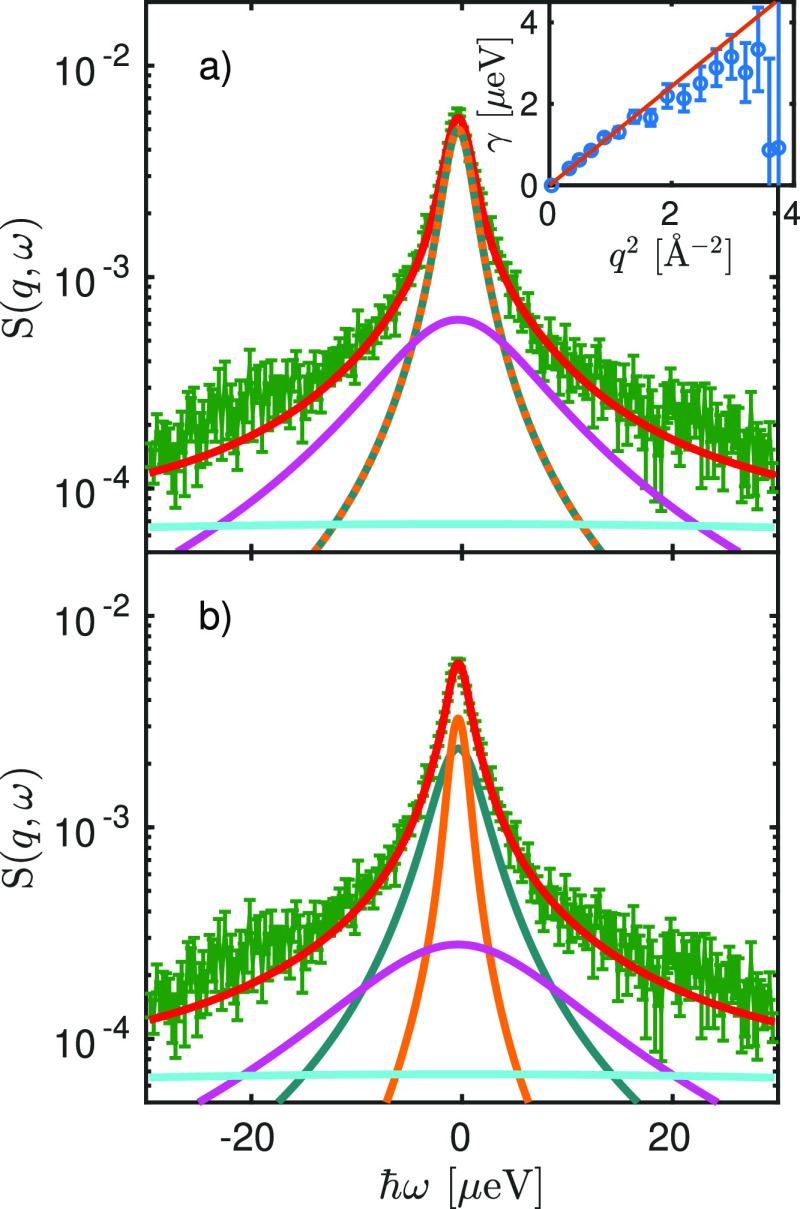
Spectra with its statistical errors at *q* = 1 Å^–1^ with *c*_BSA_ = 200 mg/mL and *c*_Ig_ = 100 mg/mL at *T* = 295 K displayed in green. (a) Total fit based on eq 3, the averaged center-of-mass, averaged internal, and the solvent contribution are displayed as red, blue-orange dotted, magenta, and cyan lines, respectively. Inset: Width of the Lorentzian function describing the averaged center-of-mass diffusion as a function of *q*^2^. The solid line represents a fit of γ = *D*_av_*q*^2^. (b) Fit result based on eq 5 (red line), containing the contribution of the apparent center-of-mass diffusion of BSA (blue line) and of Ig (orange line), the contribution of the averaged internal diffusion (magenta line), and the contribution of the solvent (cyan line).

A representative fit result is shown in Figure 1a. The inset of Figure 1a depicts the fitted width γ versus *q*^2^. Although the fit averages over the two apparent diffusion coefficients associated with BSA and Ig, respectively, the relationship γ = *D*_exp_^(av)^*q*^2^ (solid line) is not imposed, yet it arises naturally from the *q*-wise fits. This indicates that on the timescale ranging from some tens of picoseconds to some nanoseconds accessible by IN16B, the averaged center-of-mass diffusion of Ig and BSA undergoes a simple Fickian diffusion with an averaged apparent diffusion coefficient *D*_exp_^(av)^, comprising the translational and rotational diffusion contributions of both Ig and BSA. Due to the limited energy range |ℏ*ω*| ≤ 30 μeV of IN16B, the fitted γ(*q*) seemingly deviates from this relationship at the highest *q* (inset of Figure 1a), which can be attributed to this sampling and intensity limitation and is absent when spectrometers with a larger energy range are employed.^64,65^

**Figure 2:**
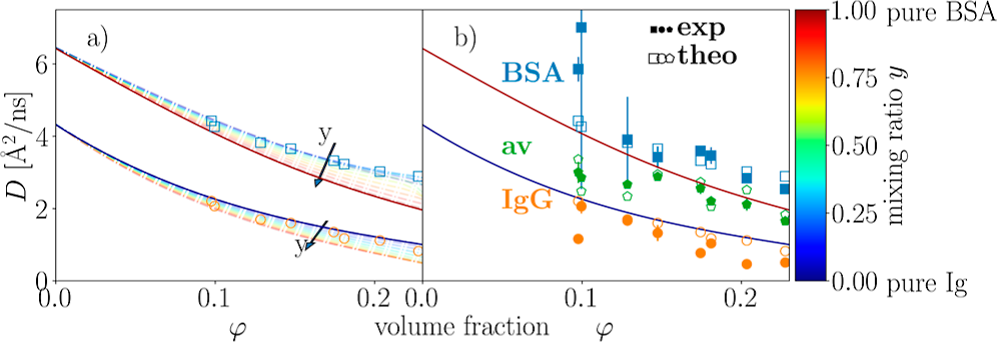
Diffusion coefficients as a function of the total volume fraction φ. The red and blue solid lines indicate the parametrization of the experimentally determined φ dependence of the short time self-diffusion coefficient of pure BSA and Ig, respectively. (a) Dotted and dashed-dotted lines represent the short time self-diffusion of BSA and Ig, respectively, calculated as explained in the main text based on Wang and Brady^38^ in mixtures containing both proteins with the mixing ratio *y* color-coded in the colorbar on the right. The calculated values for the samples investigated are additionally represented by open orange circles and open blue squares for pure Ig and pure BSA, respectively. (b) Averaged calculated diffusion coefficient in the mixture is displayed as open green pentagrams for the sample conditions investigated. The filled symbols represent the experimentally determined diffusion coefficients *D* of the average (green pentagons), of Ig (orange circles), and of BSA (blue squares) in the mixtures. Note that the confidence bounds on the fits depend on the mixing ratio. In samples with a very low volume fraction of one component, these result in large error bars on the symbols.

The average apparent diffusion coefficients *D*_exp_^(av)^ obtained from samples measured at *T* = 295 K (green pentagons), as well as the volume fraction dependencies of the diffusion coefficients of the pure protein solutions determined earlier [BSA (red solid line)^31^ and Ig (blue solid line)^44^], are shown as a function of the total volume fraction of the system φ = φ_BSA_ + φ_Ig_ in Figure 2b. In this case, φ_Ig_ = *c*_Ig_·ν_Ig_ and φ_BSA_ = *c*_BSA_·ν_BSA_ are the volume fractions calculated based on the partial specific volume of Ig ν_Ig_ = 0.739 mL/g (ref (66)) and BSA ν_BSA_ = 0.735 mL/g (ref (67)), respectively. Overviews of the measured samples and the corresponding parameters are given in Table S1 and Table S2. The dependence of the averaged apparent diffusion coefficient *D*_exp_^(av)^ on φ (green pentagon symbols in Figure 2b) is not monotonic because the mixing ratio *y* = φ_BSA_/φ varies. Nevertheless, the observed average diffusion coefficients *D*_exp_^(av)^(φ,*y*) are within the limits given by *D*^(Ig)^(*φ*) and *D*^(BSA)^(φ) of the pure Ig and BSA solution, respectively.

### 3.2 Comparison with the Calculated Average Center-Of-Mass Diffusion

In Figure 2a, the theoretical diffusion coefficients *D*_theo_^(BSA)^ and *D*_theo_^(Ig)^ for the individual components (BSA and Ig) in the mixture, calculated as explained in Section 2.6, are displayed for different mixing ratios *y* as a function of the total volume fraction φ (dash-dotted lines). The apparent diffusion coefficients for Ig in the mixture *D*_theo_^(Ig)^(φ) are lower than the ones for Ig in the monodisperse solution *D*^(Ig)^(φ). In contrast, for the smaller protein BSA, the apparent diffusion coefficient in the presence of Ig *D*_theo_^(BSA)^(φ) is increased compared to the monodisperse case *D*^(BSA)^(φ). This observation is in agreement with several previous studies.^38,39^ For the sample conditions investigated, the theoretical diffusion coefficients are displayed in addition as open symbols in Figure 2a (note that the mixing ratio *y* for each symbol is different). Based on these calculated theoretical diffusion coefficients, the averaged diffusion coefficient is calculated using the same approach as for the experimental data by approximating the sum of the two weighted Lorentzian functions with one single Lorentzian function. The calculated averaged diffusion coefficient, displayed as open green symbols in Figure 2b, agrees well with the experimentally determined ones displayed as filled green pentagons.

### 3.3 Individual Center-Of-Mass Diffusion of BSA and Ig

In Section 3.2, the deviation of the calculated diffusion coefficient of the protein in the mixture from the diffusion coefficient at the same volume fraction for the single-component solution is described. To investigate the diffusion coefficients of BSA and Ig simultaneously, the experimental data were reanalyzed as explained in Section 2.5. Figure 1b displays an example spectrum with the fit of eq 5 extracting simultaneously the apparent global diffusion coefficients of BSA and Ig in the mixtures. The diffusion coefficients are displayed for the different samples in Figure 2b as filled blue squares and filled orange circles for BSA and Ig, respectively. As it can be seen, according to this analysis, the experimental diffusion coefficients in the mixtures significantly deviate from the values in the pure protein solutions at the same φ (solid lines). While the larger protein Ig is slowed down due to the presence of BSA at the same total volume fraction φ (i.e., *D*^(Ig)^(*φ*)>*D*_exp_^(Ig)^(*φ*)), the smaller protein BSA is accelerated (i.e., *D*^(BSA)^(*φ*) < *D*_exp_^(BSA)^(*φ*)). Even though the bicomponent model seems to provide physically reasonable results, one must be aware that the data quality even under optimized experimental conditions would not allow such a separation without applying the existing knowledge on the sample composition. Overall, there is a risk of overinterpreting the data. Nevertheless, the χ_ν_^2^ method employed here, that is, the maximum likelihood method for Poisson-distributed data, represents the best available statistical test to our knowledge.^68^ The fact that we obtain reasonable confidence intervals stresses that we have not employed a model with redundant fit parameters.

**Figure 3:**
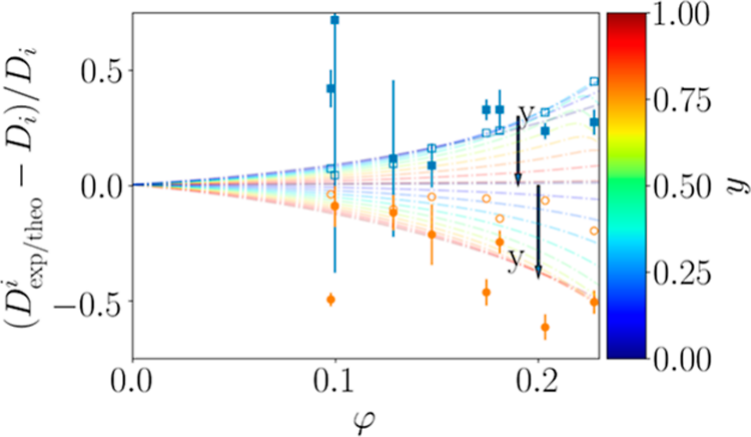
Relative change in the diffusion coefficients  as a function of the volume fraction φ. The corresponding mixing ratio *y* is color-coded. Dashed dotted lines represent the deviations predicted by Brady and Wang. The corresponding theoretical values for the specific sample conditions are additionally displayed as open blue squares and open orange circles for BSA and Ig, respectively. Blue and orange filled symbols represent the experimentally determined percentage deviations of BSA and Ig, respectively, in the mixture compared to the monodisperse solution. Note that the confidence bounds on the fits depend on the mixing ratio. In samples with a very low concentration of one component, these result in large error bars on the symbols.

To investigate the dependence on the mixing ratio *y*, the relative change in the diffusion coefficients
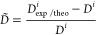
10for *i* representing BSA and Ig is shown for both proteins in Figure 3 as a function of φ for different *y*.

A systematic trend is seen for both proteins within the investigated range of *y*. Here, the bigger protein Ig is slowed down, while the smaller protein present in solution is accelerated. It should be mentioned that approaching the limits *y* = 0 and *y* = 1, the diffusion coefficients of the proteins in the mixture have to approach the values for the respective pure solutions
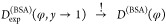
11
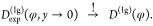
12

**Figure 4:**
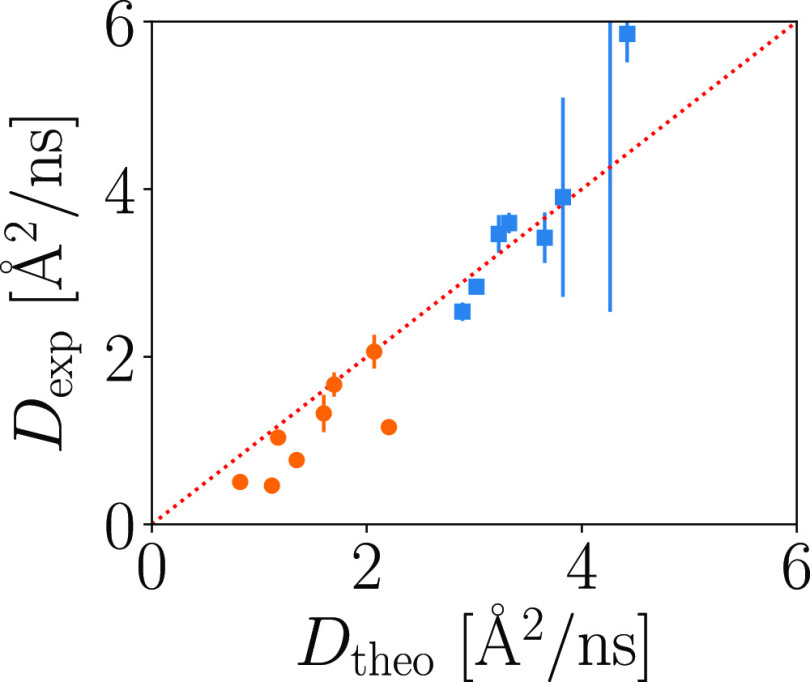
Experimentally determined diffusion coefficients *D*_exp_^*i*^ as a function of the corresponding calculated diffusion coefficients *D*_theo_^*i*^ according to the model by Wang and Brady^38^ as explained in the text. Filled blue squares and filled orange points represent the experimental diffusion coefficients of BSA and Ig, respectively. Note that the confidence bounds on the fits depend on the mixing ratio. In samples with a very low volume fraction of one component, these result in large error bars on the symbols.

The good agreement between the experimental *D*_exp_^*i*^ and the theoretically calculated *D*_theo_^*i*^ (Section 2.6) is confirmed in Figure 4, where the individual experimental diffusion coefficients for each component *D*_exp_^*i*^ are plotted as a function of the calculated diffusion coefficient *D*_theo_^*i*^ for each sample (symbols). We note that Ig is not spherical which might cause a small systematic error in the calculation of the apparent diffusion coefficient *D*_theo_^*i*^ based on the translational and rotational diffusion coefficient. The bisector marked by the dotted line would correspond to a perfect agreement of the result from the fit of the measured spectra in terms of the bidisperse model (eq 5) with the result from the calculation according to Wang and Brady.^38^ This figure also illustrates the distinct diffusion coefficients of the two components, namely, Ig and BSA, in the mixture (orange circles and blue squares, respectively). We have not considered the nonisotropic shape of the Ig proteins and their possible patchy interactions, although both anisotropy and charge-mediated interactions have been shown to influence protein–protein interactions in the short-time limit.^9,69^ We attribute the high consistency of our results with a picture of purely hydrodynamic interactions to the fact that our Ig is polyclonal, thus featuring many different charge patterns and resulting in no net large effect of the overall patchy interactions, and to self-buffering of the protein solutions at high concentrations.^62^

**Figure 5:**
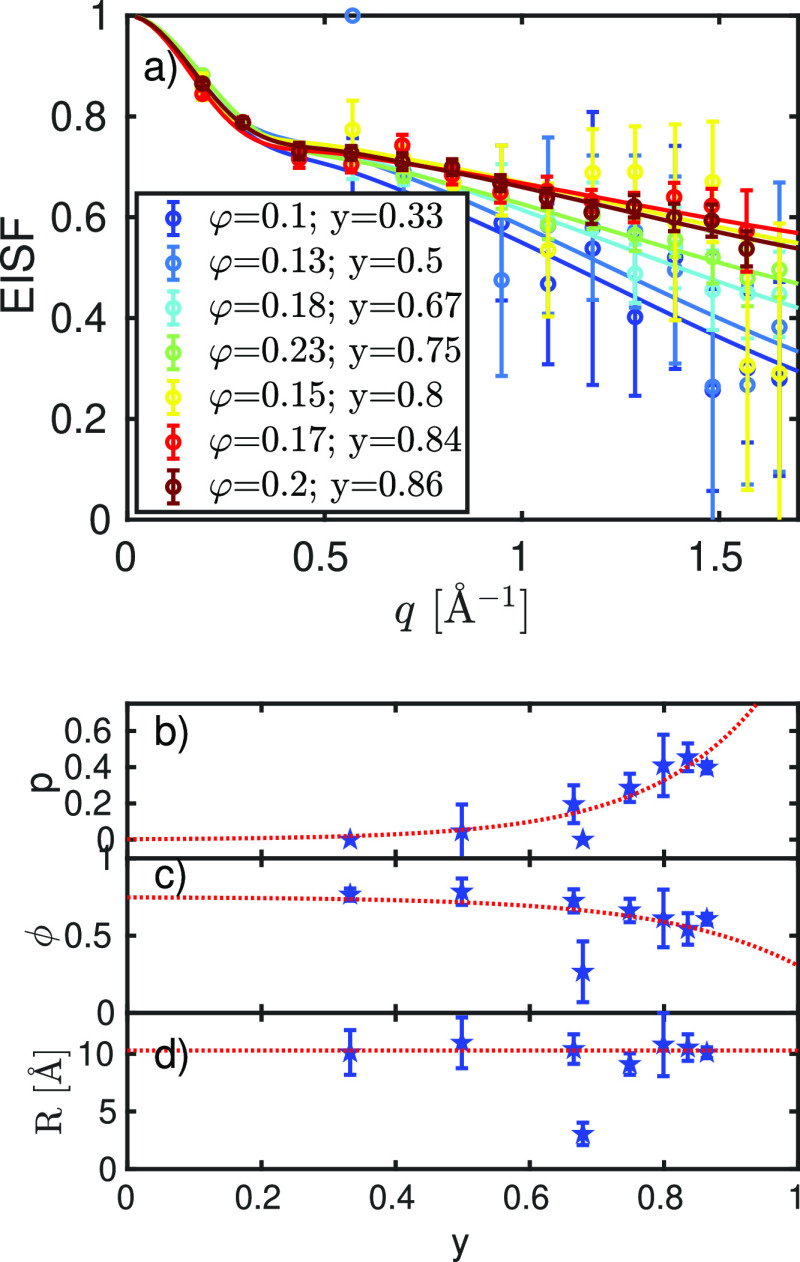
Averaged EISF as a function of *q* of the different samples investigated. Fits of eq 13 are shown as solid lines. Figures b-d represent the fit parameters *p*, ϕ, and *R* as a function of the mixing ratio *y*. Red dashed lines are guides to the eye.

Due to the restricted energy range of the spectrometer, a further separation of the internal dynamics contributions, which are significantly faster than the center-of-mass diffusion, is not possible. In Figure 5, the averaged EISF is shown for the different samples investigated. We used previously established models to describe the q dependence^44^
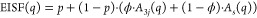
13
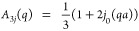
14
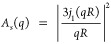
15with *a* = 1.715 Å being the averaged distance between the hydrogens in the methyl group to fit the EISF. The dependence of *p*, ϕ, and *R* on the mixing ratio *y* is shown in Figure 5. The width of the Lorentzian describing the averaged internal diffusion is approximated by a jump diffusion model to describe the *q* dependencies. The corresponding fits and their results are shown in Figure S5 in the Supporting Information. Although the parameters for the EISF *p* and ϕ show trends as a function of *y*, the jump diffusion parameters, namely, the residence time τ and the diffusion coefficient of *D*_int_, stay nearly constant at τ ≈ 0.1 ns and *D*_int_ ≈ 100 Å^2^/ns, respectively, as shown in Figure S5. Also, *R* remains nearly constant close to the value of 10 Å. Therefore, average protein dynamics between BSA and Ig as seen by QENS appear to be rather similar. Otherwise, one would see random jumps in the internal dynamics when the mixing ratio is varied.

From a biological point of view, the observation presented in this work is particularly interesting for cases in which reactions are limited or enhanced by diffusion and crowded settings.^70^ Examples are red blood cells, where the slow long-time diffusion of concentrated hemoglobin was recently suggested to be crucial in maximizing the oxygen capture at the cell level during the time spent near the alveolar sac^71^ or the DNA replication, where nucleotides are diffusing toward the DNA polymerase which are then used to complement the second new complementary DNA strand.^72^

## 4 Conclusions

Using quasi-elastic neutron scattering, we have probed the self-diffusion of proteins in crowded bidisperse suspensions at mixing compositions which were precisely set by the design of the experiment. This design constitutes a minimal model system to study effects of polydispersity in a controllable manner. We have provided a benchmark for the analysis of high-resolution quasi-elastic neutron scattering spectra by accounting for two distinct proteins in aqueous solution in our model scattering function. We successfully obtained the apparent global center-of-mass diffusion coefficients for both proteins. In addition, the averaged internal diffusion contributions were separated from the signal. Within the experimental accuracy, we find quantitative agreement between the corresponding colloid model system of bidisperse hard spheres and the experimental results for the protein center-of-mass short-time self-diffusion. In particular, there are significant deviations of the diffusion for each component in the mixture when compared to the monodisperse case both in experiment and colloid theory. This short-time diffusion on the nanosecond time scale, where hydrodynamic interactions dominate, constitutes an important quantity for both the cellular function and for the calibration of long-time diffusion. Our results illustrate the predictive power of colloid hard sphere models for protein diffusion on the observation scale of short-time diffusion for the situation of two distinct protein sizes. These results contribute to a better understanding of the role of macromolecular polydispersity in living systems. This polydispersity and resulting dispersion of diffusion rates influence the diffusive transport in living cells and, thus, their function.

## Supporting Information Available

Details on the calculation of the theoretical translational and rotational diffusion coefficient and on the calculation of the apparent diffusion coefficients and overview of the measured samples (PDF)

## References

[ref1] Maheshwari A. J.; Sunol A. M.; Gonzalez E.; Endy D.; Zia R. N. Colloidal hydrodynamics of biological cells: A frontier spanning two fields. Phys. Rev. Fluids 2019, 4, 110506 10.1103/physrevfluids.4.110506.

[ref2] Witzel P.; Götz M.; Lanoiselée Y.; Franosch T.; Grebenkov D. S.; Heinrich D. Heterogeneities shape passive intracellular transport. Biophys. J. 2019, 117, 203–213. 10.1016/j.bpj.2019.06.009. 31278001 PMC6700759

[ref3] Cho H. W.; Kwon G.; Sung B. J.; Yethiraj A. Effect of polydispersity on diffusion in random obstacle matrices. Phys. Rev. Lett. 2012, 109, 155901 10.1103/physrevlett.109.155901. 23102336

[ref4] Gonzalez E.; Aponte-Rivera C.; Zia R. N. Impact of polydispersity and confinement on diffusion in hydrodynamically interacting colloidal suspensions. J. Fluid Mech. 2021, 925, A35 10.1017/jfm.2021.563.

[ref5] Ilker E.; Castellana M.; Joanny J.-F. Long-time diffusion and energy transfer in polydisperse mixtures of particles with different temperatures. Phys. Rev. Res. 2021, 3, 023207 10.1103/PhysRevResearch.3.023207.

[ref6] Ellis R. J. Macromolecular Crowding: An Important but Neglected Aspect of the Intracellular Environment. Curr. Opin. Struct. Biol. 2001, 11, 114–119. 10.1016/s0959-440x(00)00172-x. 11179900

[ref7] Höfling F.; Franosch T. Anomalous Transport in the Crowded World of Biological Cells. Rep. Prog. Phys. 2013, 76, 046602 10.1088/0034-4885/76/4/046602. 23481518

[ref8] Gupta S.; Biehl R.; Sill C.; Allgaier J.; Sharp M.; Ohl M.; Richter D. Protein Entrapment in Polymeric Mesh: Diffusion in Crowded Environment with Fast Process on Short Scales. Macromolecules 2016, 49, 1941–1949. 10.1021/acs.macromol.5b02281.

[ref9] Bucciarelli S.; Myung J. S.; Farago B.; Das S.; Vliegenthart G. A.; Holderer O.; Winkler R. G.; Schurtenberger P.; Gompper G.; Stradner A. Dramatic influence of patchy attractions on short-time protein diffusion under crowded conditions. Sci. Adv. 2016, 2, e1601432 10.1126/sciadv.1601432. 27957539 PMC5142800

[ref10] Vodnala P.; Karunaratne N.; Lurio L.; Thurston G. M.; Gaillard E.; Narayanan S.; Sandy A.; Zhang Q.; Dufresne E. M.; et al. Hard-sphere-like dynamics in highly concentrated alpha-crystallin suspensions. Phys. Rev. E 2018, 97, 020601 10.1103/PhysRevE.97.020601. 29548072

[ref11] Ostrowska N.; Feig M.; Trylska J. Modeling crowded environment in molecular simulations. Front. Mol. Biosci. 2019, 6, 86 10.3389/fmolb.2019.00086. 31572730 PMC6749006

[ref12] Fagerberg E.; Lenton S.; Nylander T.; Seydel T.; Skepö M. Self-Diffusive Properties of the Intrinsically Disordered Protein Histatin 5 and the Impact of Crowding Thereon: A Combined Neutron Spectroscopy and Molecular Dynamics Simulation Study. J. Phys. Chem. B 2022, 126, 789–801. 10.1021/acs.jpcb.1c08976. 35044776 PMC8819652

[ref13] Makarov V.; Pettitt B.; Feig M. Solvation and Hydration of Proteins and Nucleic Acids: A Theoretical View of Simulation and Experiment. Acc. Chem. Res. 2002, 35, 376–384. 10.1021/ar0100273. 12069622

[ref14] Nawrocki G.; Wang P.-h.; Yu I.; Sugita Y.; Feig M. Slow-Down in Diffusion in Crowded Protein Solutions Correlates with Transient Cluster Formation. J. Phys. Chem. B 2017, 121, 11072–11084. 10.1021/acs.jpcb.7b08785. 29151345 PMC5951686

[ref15] Feig M.; Yu I.; Wang P.-h.; Nawrocki G.; Sugita Y. Crowding in Cellular Environments at an Atomistic Level from Computer Simulations. J. Phys. Chem. B 2017, 121, 8009–8025. 10.1021/acs.jpcb.7b03570. 28666087 PMC5582368

[ref16] Doster W.; Longeville S. Microscopic Diffusion and Hydrodynamic Interactions of Hemoglobin in Red Blood Cells. Biophys. J. 2007, 93, 1360–1368. 10.1529/biophysj.106.097956. 17513357 PMC1929019

[ref17] Stadler A.; Digel I.; Artmann G. M.; Embs J. P.; Zaccai G.; Büldt G. Hemoglobin Dynamics in Red Blood Cells: Correlation to Body Temperature. Biophys. J. 2008, 95, 5449–5461. 10.1529/biophysj.108.138040. 18708462 PMC2586580

[ref18] Jasnin M.; Moulin M.; Haertlein M.; Zaccai G.; Tehei M. In Vivo Measurement of Internal and Global Macromolecular Motions in Escherichia Coli. Biophys. J. 2008, 95, 857–864. 10.1529/biophysj.107.124420. 18359790 PMC2440467

[ref19] Anunciado D. B.; Nyugen V. P.; Hurst G. B.; Doktycz M. J.; Urban V.; Langan P.; Mamontov E.; O’Neill H. In Vivo Protein Dynamics on the Nanometer Length Scale and Nanosecond Time Scale. J. Phys. Chem. Lett. 2017, 8, 1899–1904. 10.1021/acs.jpclett.7b00399. 28388043

[ref20] Mamontov E. Microscopic diffusion processes measured in living planarians. Sci. Rep. 2018, 8, 4190 10.1038/s41598-018-22643-9. 29520078 PMC5843606

[ref21] Roos M.; Link S.; Balbach J.; Krushelnitsky A.; Saalwächter K. NMR-detected Brownian dynamics of αB-crystallin over a wide range of concentrations. Biophys. J. 2015, 108, 98–106. 10.1016/j.bpj.2014.11.1858. 25564856 PMC4286604

[ref22] Roos M.; Ott M.; Hofmann M.; Link S.; Rössler E.; Balbach J.; Krushelnitsky A.; Saalwächter K. Coupling and decoupling of rotational and translational diffusion of proteins under crowding conditions. J. Am. Chem. Soc. 2016, 138, 10365–10372. 10.1021/jacs.6b06615. 27434647

[ref23] Nikitin A. A.; Yurenya A. Y.; Gabbasov R. R.; Cherepanov V. M.; Polikarpov M. A.; Chuev M. A.; Majouga A. G.; Panchenko V. Y.; Abakumov M. A. Effects of Macromolecular Crowding on Nanoparticle Diffusion: New Insights from Mossbauer Spectroscopy. J. Phys. Chem. Lett. 2021, 12, 6804–6811. 10.1021/acs.jpclett.1c01984. 34270251

[ref24] Banks D. S.; Fradin C. Anomalous diffusion of proteins due to molecular crowding. Biophys. J. 2005, 89, 2960–2971. 10.1529/biophysj.104.051078. 16113107 PMC1366794

[ref25] Bacia K.; Kim S. A.; Schwille P. Fluorescence cross-correlation spectroscopy in living cells. Nat. Methods 2006, 3, 83–89. 10.1038/nmeth822. 16432516

[ref26] Kalwarczyk T.; Kwapiszewska K.; Szczepanski K.; Sozanski K.; Szymanski J.; Michalska B.; Patalas-Krawczyk P.; Duszynski J.; Holyst R. Apparent anomalous diffusion in the cytoplasm of human cells: the effect of probes’ polydispersity. J. Phys. Chem. B 2017, 121, 9831–9837. 10.1021/acs.jpcb.7b07158. 28956920

[ref27] Tokuyama M.; Oppenheim I. Dynamics of Hard-Sphere Suspensions. Phys. Rev. E: Stat. Phys., Plasmas, Fluids, Relat. Interdiscip. Top. 1994, 50, R16–R19. 10.1103/physreve.50.r16. 9962019

[ref28] Nägele G. On the Dynamics and Structure of Charge-Stabilized Suspensions. Phys. Rep. 1996, 272, 215–372. 10.1016/0370-1573(95)00078-x.

[ref29] Banchio A. J.; Nägele G. Short-Time Transport Properties in Dense Suspensions: From Neutral to Charge-Stabilized Colloidal Spheres. J. Chem. Phys. 2008, 128, 104903 10.1063/1.2868773. 18345924

[ref30] Le Coeur C.; Longeville S. Microscopic Protein Diffusion at High Concentration by Neutron Spin-Echo Spectroscopy. Chem. Phys. 2008, 345, 298–304. 10.1016/j.chemphys.2007.09.042.

[ref31] Roosen-Runge F.; Hennig M.; Zhang F.; Jacobs R. M. J.; Sztucki M.; Schober H.; Seydel T.; Schreiber F. Protein Self-Diffusion in Crowded Solutions. Proc. Natl. Acad. Sci. U.S.A. 2011, 108, 11815–11820. 10.1073/pnas.1107287108. 21730176 PMC3142006

[ref32] Rothe M.; Gruber T.; Gröger S.; Balbach J.; Saalwächter K.; Roos M. Transient binding accounts for apparent violation of the generalized Stokes–Einstein relation in crowded protein solutions. Phys. Chem. Chem. Phys. 2016, 18, 18006–18014. 10.1039/c6cp01056c. 27326536

[ref33] Ando T.; Skolnick J. Crowding and Hydrodynamic Interactions Likely Dominate In Vivo Macromolecular Motion. Proc. Natl. Acad. Sci. U.S.A. 2010, 107, 18457–18462. 10.1073/pnas.1011354107. 20937902 PMC2973006

[ref34] Cichocki B.; Felderhof B. Long-time self-diffusion coefficient and zero-frequency viscosity of dilute suspensions of spherical Brownian particles. J. Chem. Phys. 1988, 89, 3705–3709. 10.1063/1.454891.

[ref35] Brady J. F. The long-time self-diffusivity in concentrated colloidal dispersions. J. Fluid Mech. 1994, 272, 109–134. 10.1017/s0022112094004404.

[ref36] Zahn K.; Méndez-Alcaraz J. M.; Maret G. Hydrodynamic Interactions May Enhance the Self-Diffusion of Colloidal Particles. Phys. Rev. Lett. 1997, 79, 175–178. 10.1103/physrevlett.79.175.

[ref37] Bleibel J.; Domínguez A.; Günther F.; Harting J.; Oettel M. Hydrodynamic interactions induce anomalous diffusion under partial confinement. Soft Matter 2014, 10, 2945–2948. 10.1039/c3sm53043d. 24647326

[ref38] Wang M.; Brady J. F. Short-time transport properties of bidisperse suspensions and porous media: A Stokesian dynamics study. J. Chem. Phys. 2015, 142, 094901 10.1063/1.4913518. 25747100

[ref39] Grimaldo M.; Lopez H.; Beck C.; Roosen-Runge F.; Moulin M.; Devos J. M.; Laux V.; Härtlein M.; Da Vela S.; Schweins R.; et al. Protein short-time diffusion in a naturally crowded environment. J. Phys. Chem. Lett. 2019, 10, 1709–1715. 10.1021/acs.jpclett.9b00345. 30897330

[ref40] Girelli A.; Beck C.; Bäuerle F.; Matsarskaia O.; Maier R.; Zhang F.; Wu B.; Lang C.; Czakkel O.; Seydel T.; et al. Molecular flexibility of antibodies preserved even in dense phase after macroscopic phase separation. Mol. Pharm. 2021, 18, 4162 10.1021/acs.molpharmaceut.1c00555. 34637319 PMC8564753

[ref41] Cisse A.; Schachner-Nedherer A.-L.; Appel M.; Beck C.; Ollivier J.; Leitinger G.; Prassl R.; Kornmueller K.; Peters J. Dynamics of Apolipoprotein B-100 in Interaction with Detergent Probed by Incoherent Neutron Scattering. J. Phys. Chem. Lett. 2021, 12, 12402–12410. 10.1021/acs.jpclett.1c03141. 34939807

[ref42] Hay F. C.; Westwood O. M. R. Practical Immunology; Wiley: New York, 2002.

[ref43] Babcock J. J.; Brancaleon L. Bovine serum albumin oligomers in the E- and B-forms at low protein concentration and ionic strength. Int. J. Biol. Macromol. 2013, 53, 42–53. 10.1016/j.ijbiomac.2012.10.030. 23148944 PMC3605742

[ref44] Grimaldo M.; Roosen-Runge F.; Zhang F.; Seydel T.; Schreiber F. Diffusion and Dynamics of γ-Globulin in Crowded Aqueous Solutions. J. Phys. Chem. B 2014, 118, 7203–7209. 10.1021/jp504135z. 24871685

[ref45] Beck C.; Grimaldo M.; Roosen-Runge F.; Braun M. K.; Zhang F.; Schreiber F.; Seydel T. Nanosecond Tracer Diffusion as a Probe of the Solution Structure and Molecular Mobility of Protein Assemblies: The Case of Ovalbumin. J. Phys. Chem. B 2018, 122, 8343–8350. 10.1021/acs.jpcb.8b04349. 30106587

[ref46] Seydel T.; Da Vela S.; Feustel M.; Grimaldo M.; Roosen-Runge F.; Schreiber F.; Zhang F. Crowding in Ternary Protein Solutions, 2014,*ILL data*, DOI: https://doi.org/10.5291/ILL-DATA.9-13-526.

[ref47] Grimaldo M.; Beck C.; Da Vela S.; Feustel M.; Roosen-Runge F.; Schreiber F.; Seydel T.; Sohmen B.; Zhang F. External Crowding in Protein Solutions, 2015, *ILL data*, DOI: https://doi.org/10.5291/ILL-DATA.8-04-759.

[ref48] Frick B.; Mamontov E.; Eijck L. V.; Seydel T. Recent Backscattering Instrument Developments at the ILL and SNS. Z. Phys. Chem. 2010, 224, 33–60. 10.1524/zpch.2010.6091.

[ref49] Hennig M.; Frick B.; Seydel T. Optimum Velocity of a Phase-Space Transformer for Cold-Neutron Backscattering Spectroscopy. J. Appl. Crystallogr. 2011, 44, 467–472. 10.1107/s0021889811013227.

[ref50] Van Rossum G.; Drake F. L. Python 3 Reference Manual; CreateSpace: Scotts Valley, CA, 2009.

[ref51] Grimaldo M.; Roosen-Runge F.; Zhang F.; Schreiber F.; Seydel T. Dynamics of proteins in solution. Q. Rev. Biophys. 2019, 52, E7 10.1017/s0033583519000027.

[ref52] Grimaldo M.; Roosen-Runge F.; Jalarvo N.; Zamponi M.; Zanini F.; Hennig M.; Zhang F.; Schreiber F.; Seydel T. High-Resolution Neutron Spectroscopy on Protein Solution Samples. EPJ Web of Conferences 2015, 83, 02005 10.1051/epjconf/20158302005.

[ref53] Beck C.; Grimaldo M.; Braun M. K.; Bühl L.; Matsarskaia O.; Jalarvo N. H.; Zhang F.; Roosen-Runge F.; Schreiber F.; Seydel T. Temperature and salt controlled tuning of protein clusters. Soft Matter 2021, 17, 8506–8516. 10.1039/d1sm00418b. 34490428

[ref54] Sohmen B.; Beck C.; Seydel T.; Hoffmann I.; Hermann B.; Nüesch M.; Grimaldo M.; Schreiber F.; Wolf S.; Roosen-Runge F.; Nanosecond structural dynamics of the chaperone Hsp90. 2021, arXiv:2110.10483.

[ref55] Harris L.; Larson S.; Hasel K.; McPherson A. Structure of Immunoglobulin; Protein Data Bank, 1997.

[ref56] Bujacz A.; Bujacz G. Crystal Structure of Bovine Serum Albumin; Protein data bank, 2012.

[ref57] Sears V. F. Neutron Scattering Lengths and Cross Sections. Neutron News 1992, 3, 26–37. 10.1080/10448639208218770.

[ref58] Jacrot B. The Study of Biological Structures by Neutron Scattering From Solution. Rep. Prog. Phys. 1976, 39, 911 10.1088/0034-4885/39/10/001.

[ref59] Ameseder F.; Radulescu A.; Holderer O.; Falus P.; Richter D.; Stadler A. M. Relevance of Internal Friction and Structural Constraints for the Dynamics of Denatured Bovine Serum Albumin. J. Phys. Chem. Lett. 2018, 9, 2469–2473. 10.1021/acs.jpclett.8b00825. 29688725

[ref60] Ameseder F.; Radulescu A.; Khaneft M.; Lohstroh W.; Stadler A. M. Homogeneous and heterogeneous dynamics in native and denatured bovine serum albumin. Phys. Chem. Chem. Phys. 2018, 20, 5128–5139. 10.1039/c7cp08292d. 29392269

[ref61] Jeffries C. M.; Graewert M. A.; Blanchet C. E.; Langley D. B.; Whitten A. E.; Svergun D. I. Preparing monodisperse macromolecular samples for successful biological small-angle X-ray and neutron-scattering experiments. Nat. Protoc. 2016, 11, 2122–2153. 10.1038/nprot.2016.113. 27711050 PMC5402874

[ref62] Da Vela S.; Roosen-Runge F.; Skoda M. W.; Jacobs R. M.; Seydel T.; Frielinghaus H.; Sztucki M.; Schweins R.; Zhang F.; Schreiber F. Effective interactions and colloidal stability of bovine γ-globulin in solution. J. Phys. Chem. B 2017, 121, 5759–5769. 10.1021/acs.jpcb.7b03510. 28520443

[ref63] Braun M. K.; Grimaldo M.; Roosen-Runge F.; Hoffmann I.; Czakkel O.; Sztucki M.; Zhang F.; Schreiber F.; Seydel T. Crowding-Controlled Cluster Size in Concentrated Aqueous Protein Solutions: Structure, Self-and Collective Diffusion. J. Phys. Chem. Lett. 2017, 8, 2590–2596. 10.1021/acs.jpclett.7b00658. 28525282

[ref64] Grimaldo M.; Roosen-Runge F.; Hennig M.; Zanini F.; Zhang F.; Jalarvo N.; Zamponi M.; Schreiber F.; Seydel T. Hierarchical Molecular Dynamics of Bovine Serum Albumin in Concentrated Aqueous Solution Below and Above Thermal Denaturation. Phys. Chem. Chem. Phys. 2015, 17, 4645–4655. 10.1039/c4cp04944f. 25587698

[ref65] Grimaldo M.; Roosen-Runge F.; Hennig M.; Zanini F.; Zhang F.; Zamponi M.; Jalarvo N.; Schreiber F.; Seydel T. Salt-Induced Universal Slowing Down of the Short-Time Self-Diffusion of a Globular Protein in Aqueous Solution. J. Phys. Chem. Lett. 2015, 6, 2577–2582. 10.1021/acs.jpclett.5b01073. 26266736

[ref66] Jøssang T.; Feder J.; Rosenqvist E. Photon Correlation Spectroscopy of Human IgG. J. Protein Chem. 1988, 7, 165–171. 10.1007/BF01025246. 3255367

[ref67] Lee J.; Timasheff S. Partial Specific Volumes and Interactions With Solvent Components of Proteins in Guanidine Hydrochloride. Biochemistry 1974, 13, 257–265. 10.1021/bi00699a005. 4855654

[ref68] Frome E. L.; Kutner M. H.; Beauchamp J. J. Regression Analysis of Poisson-Distributed Data. J. Am. Stat. Assoc. 1973, 68, 935–940. 10.1080/01621459.1973.10481449.

[ref69] Skar-Gislinge N.; Ronti M.; Garting T.; Rischel C.; Schurtenberger P.; Zaccarelli E.; Stradner A. A Colloid Approach to Self-Assembling Antibodies. Mol. Pharm. 2019, 16, 2394–2404. 10.1021/acs.molpharmaceut.9b00019. 31059276

[ref70] Dorsaz N.; De Michele C.; Piazza F.; De Los Rios P.; Foffi G. Diffusion-limited reactions in crowded environments. Phys. Rev. Lett. 2010, 105, 120601 10.1103/physrevlett.105.120601. 20867619

[ref71] Longeville S.; Stingaciu L.-R. Hemoglobin diffusion and the dynamics of oxygen capture by red blood cells. Sci. Rep. 2017, 7, 10448 10.1038/s41598-017-09146-9. 28874711 PMC5585185

[ref72] Gelfand D. H. Taq DNA Polymerase; Palgrave Macmillan UK: London, 1989; pp 17–22.

