# Supplementary material for: Short-Time Transport Properties of Bidisperse Suspensions of Immunoglobulins and Serum Albumins Consistent with a Colloid Physics Picture
Source: J Phys Chem B. 2022 Sep 16;126(38):7400–8. doi: 10.1021/acs.jpcb.2c02380 (PMC9527755; doi:10.1021/acs.jpcb.2c02380)
Supplement: Supplementary file 1 — jp2c02380_si_001.pdf [file jp2c02380_si_001.pdf]

Supporting Information: Short-time Transport Properties of Bidisperse Suspensions of Immunoglobulins and Serum Albumins Consistent with a Colloid Physics Picture

Christian Beck[a,b,*], Marco Grimaldo[b], Hender Lopez[c], Stefano da Vela[a,d], Benedikt Sohmen[e], Fajun Zhang[a], Martin Oettel[a], Jean-Louis Barrat[f], Felix Roosen-Runge[g,*], Frank Schreiber[a], Tilo Seydel[b,*]

[a] Institut für Angewandte Physik, Universität Tübingen, Auf der Morgenstelle 10, 72076 Tübingen, Germany.

[b] Institut Max von Laue - Paul Langevin (ILL), CS 20156, F-38042 Grenoble Cedex 9, France.

[c] School of Physics and Optometric & Clinical Sciences, Technological University Dublin, Grangegorman, D07 XT95, Ireland.

[d] current address: European Molecular Biology Laboratory (EMBL), Hamburg, Germany.

[e] Institute of Physical Chemistry, University of Freiburg, Albertstrasse 21, 79104 Freiburg, Germany.

[f] LiPhy, 38402 Saint Martin d'Héres, France

[g] Department of Biomedical Sciences and Biofilms-Research Center for Biointerfaces (BRCB), Malmö University, 20506 Malmö, Sweden.

[*] email: christian.beck@uni-tuebingen.de; felix.roosen-runge@mau.se; seydel@ill.eu

1 Details on the calculation of the theoretical translational and rotational diffusion coefficient

The translational (D_t) and rotational (D_r) diffusion coefficients were calculated using the expressions reported by Wang and Brady[1]:

$$\frac{D_t^{(\alpha)}(\varphi, y)}{D_0^{(\alpha)}} = 1 + \left(\sum_\beta I_{\alpha\beta}^t \varphi_\beta\right) \times (1 + 0.1195\varphi - 0.70\varphi^2), \tag{1}$$

$$\frac{D_r^{(\alpha)}(\varphi, y)}{D_0^{(\alpha)}} = 1 + \left(\sum_\beta I_{\alpha\beta}^r \varphi_\beta\right) \times (1 + 1.1505\varphi), \tag{2}$$

where α and β denote either BSA or Ig in our model, $y = \varphi_{\mathrm{BSA}}/\varphi$, and $I_{\alpha\beta}^r$ and $I_{\alpha\beta}^t$ are coefficients which depend on the ratio of the hydrodynamic sizes. Wang and Brady[1] report these coefficients for a range of ratio of sizes, and we use an interpolation method to obtain the I's for $R_H^{Ig}/R_H^{BSA} \approx 1.527$, assuming $R_H^{Ig} = 55$ Å[2,3] and $R_H^{BSA} = 36$ Å[4]. Figure S1 shows the values of I's as a function of $\lambda_{\beta\alpha} = R_\beta/R_\alpha$ taken from[1]. Blue circles correspond to I_t while the yellow squares correspond to I_r. The continues lines correspond to the interpolated functions. The purple diamonds are the values of I_t obtained for our BSA/Ig mixtures, while the brown triangles correspond I_r.

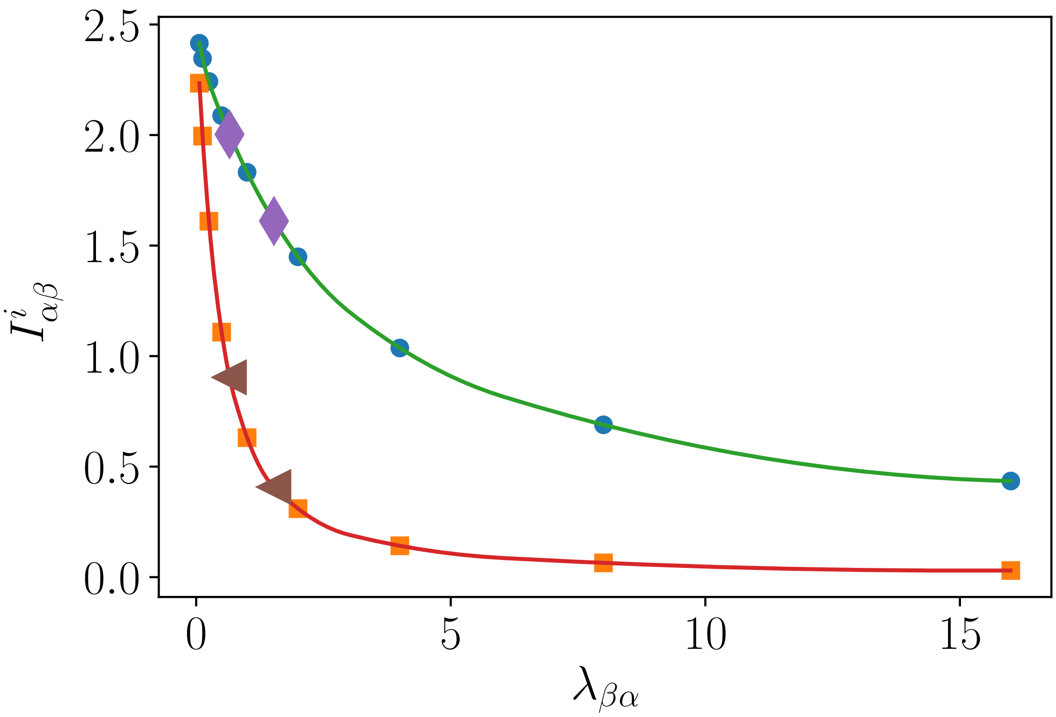

Figure S1: Determination of $I^r_{\alpha\beta}$ and $I^t_{\alpha\beta}$ for the BSA/Ig system.

2 Calculating the apparent diffusion coefficients

To calculate the apparent diffusion coefficient based on the rotational and translational diffusion coefficient in the high q limit, for each protein, the relation[5]

$$0 = \sum_{l=0}^{\infty} B_l(q) \frac{D_r l(l+1) + (D_t - D_{app}^{WB})q^2}{\left(D_r l(l+1) + (D_t + D_{app}^{WB})q^2\right)^2} \tag{3}$$

$$B_l(q) = \int_0^{\infty} dr \rho(r)(2l+1) j_l^2(qr) \tag{4}$$

is used, with D_{app}^{WB} being the apparent center-of-mass diffusion, $j_l(x)$ being the lth-order spherical Bessel function of first kind and $\rho(r)$ being the hydrogen distribution functions of Ig and BSA.The hydrogen distribution functions were approximated by homogeneously filled spheres with the corresponding radius R_H as shown in Figure S2.

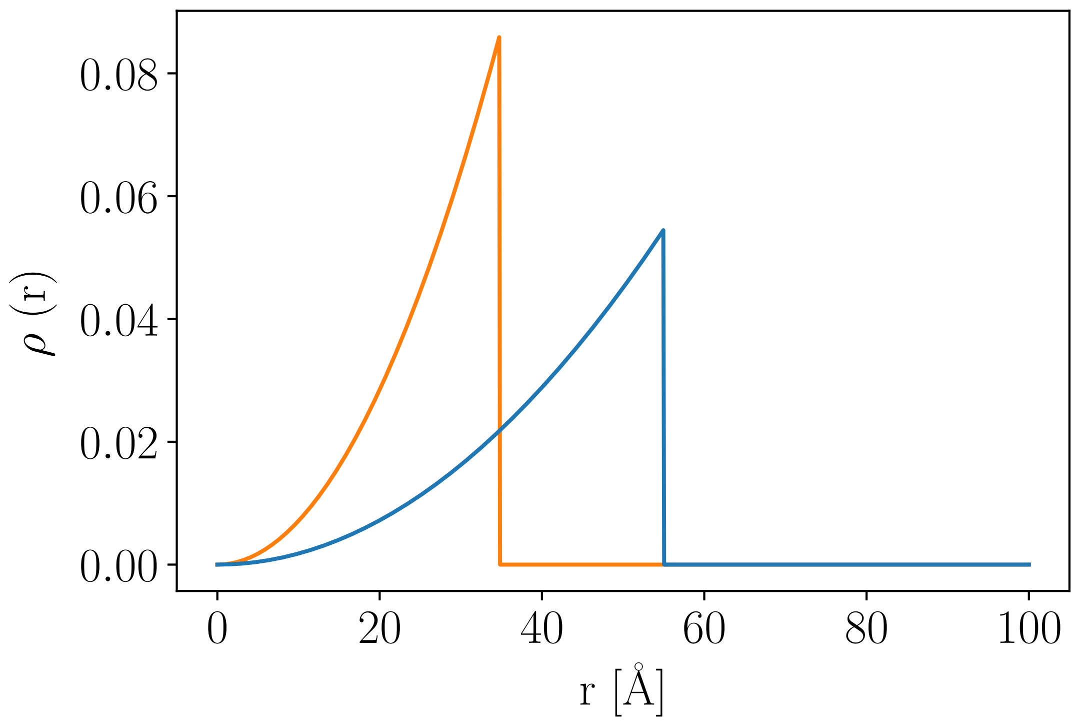

Figure S2: Hydrogen distribution function $\rho(r)$ of BSA (orange) and Ig (blue) used to calculate the apparent global diffusion coefficients.

3 Overview of the samples

Samples were prepared by weighting BSA and Ig and adding a volume D_2O. The concentrations and volume fractions were determined by $c^i = \frac{m_i}{m_{\text{BSA}} v_{\text{BSA}} + m_{\text{BSA}} v_{\text{BSA}} + V_{\text{D}_2\text{O}}}$ and $\varphi_i = c_i v_i$ with the specific volume v_i, respectively. To determine the number of proteins in the solution N, the volume fraction φ_i is multiplied by the Avogadro number N_A and divided by the molecular weight M_i with $M_{\text{BSA}} = 66.4$ kDa[6] and $M_{\text{Ig}} = 150$ kDa[7].

Table S1: Sample composition: Masses m_i, Volumes V and concentrations c^i are given in [mg], [ml] and $\left[\frac{\text{mg}}{\text{ml}}\right]$, respectively.

Experiment number	#	m_{BSA}	m_{Ig}	$V_{\text{D}_2\text{O}}$	c^{BSA}	c^{Ig}	φ_{BSA}	φ_{Ig}	$\varphi = \varphi_{\text{Ig}} + \varphi_{\text{BSA}}$	$y = \frac{\varphi_{\text{BSA}}}{\varphi}$
Exp 9-13-952	1	75	150	1.5	45.02	90.04	0.03	0.07	0.1	0.33
	2	150	150	1.5	87.15	87.15	0.06	0.06	0.13	0.50
	3	300	150	1.5	163.81	81.91	0.12	0.06	0.18	0.67
	4	450	150	1.5	231.77	77.26	0.17	0.06	0.23	0.75
	5	600	150	3.2	160.43	40.11	0.12	0.03	0.15	0.80
Exp 8-04-759	6	150	70.5	1.5	90.23	42.41	0.07	0.03	0.1	0.68
	7	360	70.5	1.5	198.16	38.81	0.15	0.03	0.17	0.84
	8	450	70.5	1.5	239	37.44	0.18	0.03	0.2	0.86

Table S2: Different diffusion coefficients and corresponding errors. For exact definition, see main text. All values are given in $\left[\frac{\text{Å}^2}{\text{ns}}\right]$.

#	D_{BSA}	D_{Ig}	D_{av}	dD_{av}	$D^{\text{BWr}}_{\text{BSA}}$	$D^{\text{BWr}}_{\text{Ig}}$	$D^{\text{2L}}_{\text{BSA}}$	$dD^{\text{2L}}_{\text{BSA}}$	$D^{\text{2L}}_{\text{Ig}}$	$dD^{\text{2L}}_{\text{Ig}}$
1	4.08	2.26	2.86	0.12	4.11	2.23	4.61	2.94	1.35	0.13
2	3.49	1.89	2.67	0.08	3.56	1.86	2.57	0.78	1.09	0.09
3	2.61	1.37	2.2	0.08	2.71	1.35	2.28	0.14	0.68	0.04
4	1.99	1.02	1.65	0.04	2.11	1.00	1.67	0.07	0.33	0.03
5	3.15	1.68	2.88	0.11	3.26	1.67	2.25	0.19	0.87	0.14
6	4.12	2.29	2.92	0.38	4.19	2.27	3.85	0.22	0.76	0.04
7	2.71	1.43	2.53	0.15	2.83	1.42	2.36	0.08	0.50	0.05
8	2.29	1.19	2.1	0.17	2.43	1.18	1.86	0.05	0.30	0.04

4 Comparison of the two fit approaches

For the different fit approaches, the fit quality was determined by calculating $\chi^2_\nu := \frac{1}{\nu} \sum_i^n \frac{(O_i - C_i)^2}{\sigma_i^2}$ with O_i, C_i and σ_i being the observations (i.e., counts per spectral channel i), calculated values from the fit model, and the variance (i.e., squared Poisson error on the spectral channel i), respectively. The degree of freedom $\nu = n - m$ is determined by the number of observations (i.e., spectral channels) n and the number of fit parameters m. The different results are shown in Figure S3 as a function of y. It can be seen that for all samples investigated, χ^2_ν is closer to unity for the two-component fit.

In Figure S4, the dependence of χ^2_ν on the global fit parameters is shown for the two different fits applied. All other fit parameters are kept in their optimum position

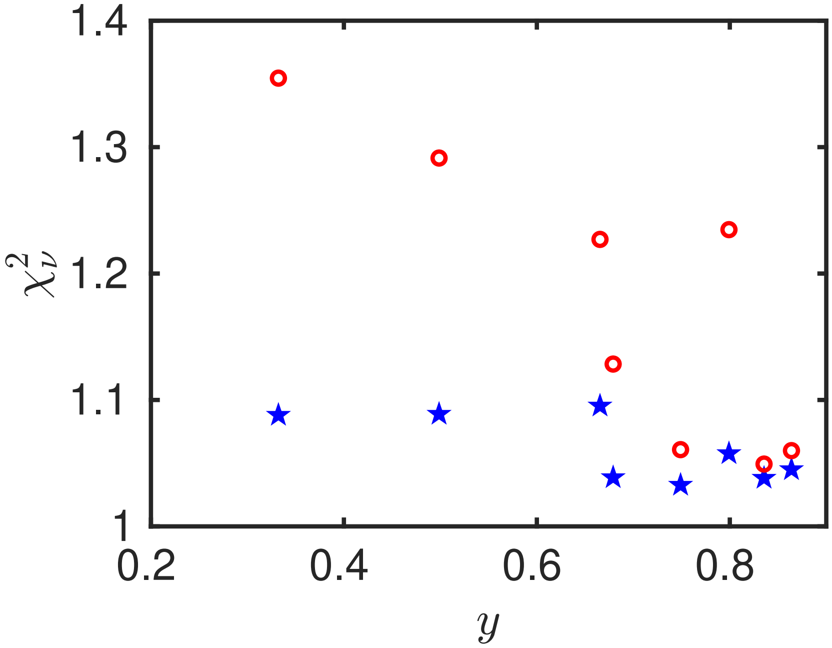

Figure S3: χ^2_ν as a function of the protein mixing ratio y. Red and blue symbols represent the fit using one averaging Lorentzian function for the averaged center of mass diffusion and two lorentzian functions describing the center of mass diffusion of BSA and Ig individually, respectively.

determined previously.

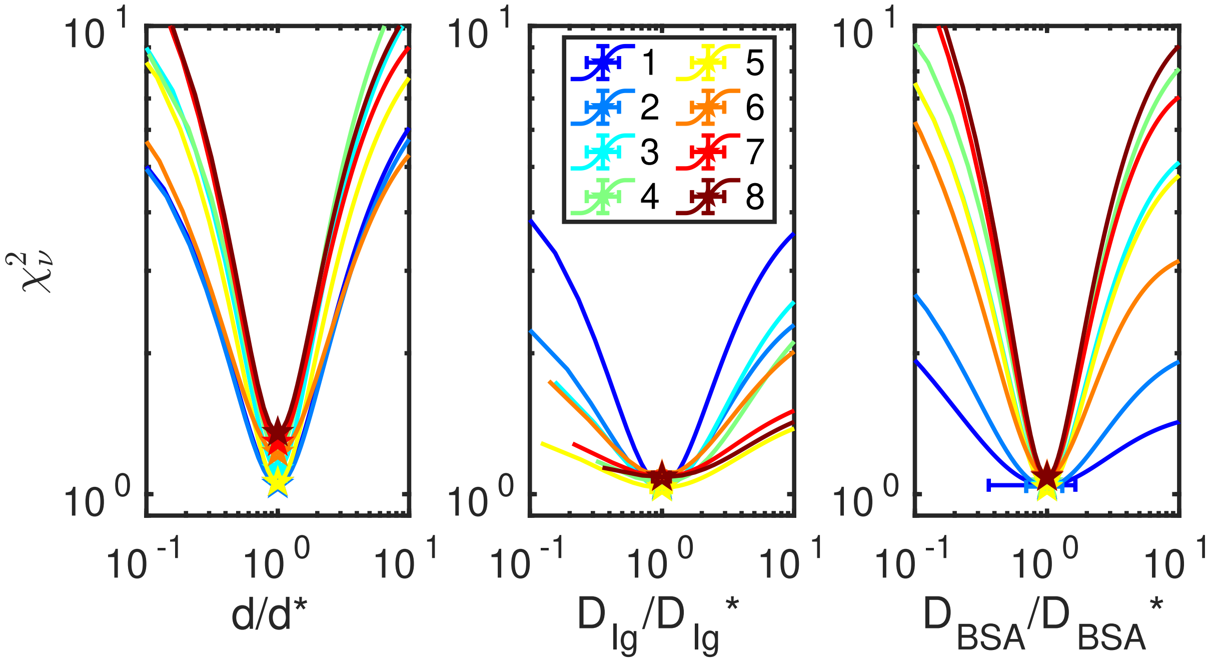

Figure S4: χ^2_ν as a function of the variation of the global fit parameters. The samples investigated are color coded and correspond to the numbers in Table S1.

5 Investigation of the averaged internal dynamics

We investigated the averaged internal diffusive processes of the samples. The width Γ averaging the internal diffusive dynamics of both types of proteins as a function of q^2 in Figure S5a. For each sample, the q dependence is approximated using the jump diffusion model[8]:

$$\Gamma = \frac{Dq^2}{1 + Dq^2\tau} \tag{5}$$

with the residence time τ and the diffusion coefficient D.

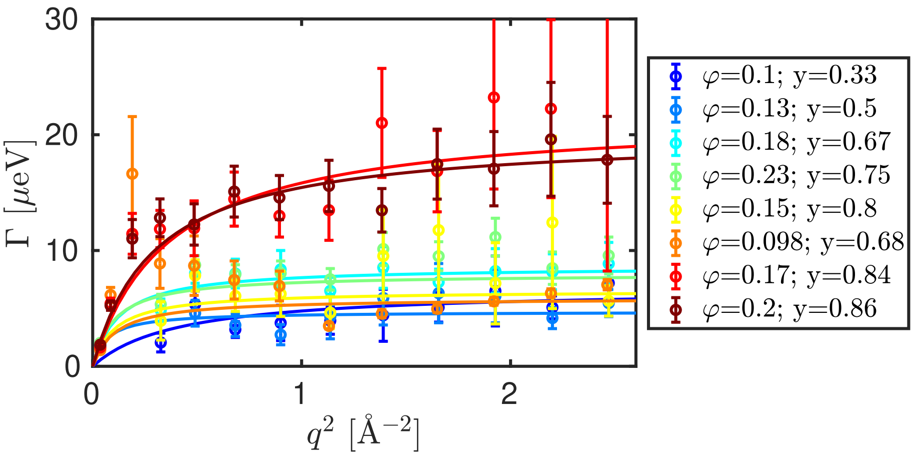

(a) Γ as a function of q^2 for different samples.

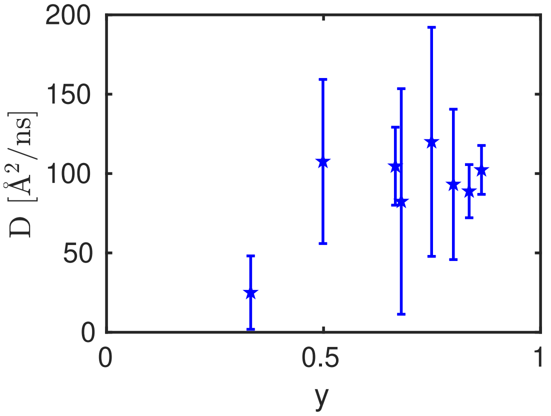

(b) Diffusion coefficient D of the jump diffusion process as a function of y.

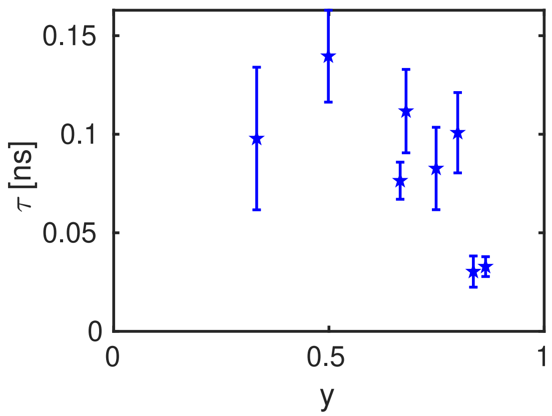

(c) Residence time τ of the jump diffusion process as a function of y.

Figure S5: Γ as well as the parameters using the jump diffusion model.

References

[1] Wang, M.; Brady, J. F. Short-time transport properties of bidisperse suspensions and porous media: A Stokesian dynamics study The Journal of Chemical Physics 2015 142, 094901.

[2] Rosenqvist, E.; Jøssang, T.; Feder, J. Thermal properties of human IgG Molecular Immunology 1987 24, 495–501.

[3] Wang, Y.; Lomakin, A.; Latypov, R. F.; Laubach, J. P.; Hideshima, T.; Richardson, P. G.; Munshi, N. C.; Anderson, K. C.; Benedek, G. B. Phase Transitions in Human IgG Solutions Journal of Chemical Physics 2013 139, 121904.

[4] Roosen-Runge, F.; Hennig, M.; Zhang, F.; Jacobs, R. M. J.; Sztucki, M.; Schober, H.; Seydel, T.; Schreiber, F. Protein Self-Diffusion in Crowded Solutions Proceedings of the National Academy of Sciences (USA) 2011 108, 11815–11820.

[5] Roosen-Runge, F.; Seydel, T. A generalized mean-squared displacement from inelastic fixed window scans of incoherent neutron scattering as a model-free indicator of anomalous diffusion confinement EPJ Web of Conferences 2015 83, 02015.

[6] Babcock, J. J.; Brancaleon, L. Bovine serum albumin oligomers in the E- and B-forms at low protein concentration and ionic strength International Journal of Biological Macromolecules 2013 53, 42–53.

[7] Hay, F. C.; Westwood, O. M. R. Practical Immunology - Wiley, New York 2002.

[8] Singwi, K. S.; Sjölander, A. Diffusive Motions in Water and Cold Neutron Scattering Physical Review 1960 119, 863–871.
